# Serpin B5 as a Potential Biomarker for the Diagnosis and Prognosis of Oral Squamous Cell Carcinoma

**DOI:** 10.7759/cureus.115829

**Published:** 2026-09-05

**Authors:** Maryam Salik, Katy Flannery, Hilal Arnouk

**Affiliations:** 1 Pathology and Laboratory Medicine, Midwestern University Chicago College of Osteopathic Medicine, Downers Grove, USA; 2 Pathology, Midwestern University Chicago College of Osteopathic Medicine, Downers Grove, USA; 3 Pathology, Illinois College of Osteopathic Medicine at The Chicago School, Chicago, USA

**Keywords:** diagnostic biomarker, head and neck cancer, molecular pathology, oral squamous cell carcinoma, precision medicine oncology, prognostic biomarker, serpin b5, tumor progression

## Abstract

Objective

Oral squamous cell carcinoma (OSCC) is a common malignancy of the head and neck region and remains to have relatively poor survival rates. Preliminary recognition of OSCC remains challenging as many patients remain asymptomatic until advanced stages, emphasizing the importance of earlier detection and accurate diagnosis. Existing diagnostic tools rely heavily on histological morphology, which has considerable variability among examiners. Serpin B5 is a serine protease inhibitor involved in vital cellular processes, including proliferation, invasion, and metastasis of malignant cells. We aimed to objectively document the expression trends in Serpin B5 in oral cancer as they relate to critical clinicopathological indicators.

Study design

Immunohistochemistry (IHC) was performed to detect Serpin B5 in the normal oral mucosa and in OSCC samples representing various clinical stages and histopathological grades. Computer-assisted analysis of IHC images was performed to consistently and objectively measure the relative abundance of Serpin B5.

Results

Serpin B5 expression analysis revealed a notable increase in OSCC tissues compared to normal oral mucosa. Furthermore, Serpin B5 expression showed significant correlations with the nodal status, clinical staging, and histopathological grading of OSCC.

Conclusion

This study documented the differential expression of Serpin B5 between normal oral mucosa and oral cancer specimens. Additionally, we found Serpin B5 expression to be significantly correlated with the histopathological grade, clinical stage, and nodal status of OSCC. The findings of this study underscore the potential application of Serpin B5 expression as a supplementary tool to aid in the diagnosis, prognosis, and monitoring of disease progression in oral cancer patients.

## Introduction

Oral squamous cell carcinoma (OSCC) is one of the most frequent head and neck neoplasms worldwide that is associated with an overall poor prognosis. The five-year survival rate remains at approximately 69% on average, depending on the stage at the time of diagnosis [1]. Lesions in the earlier stages of the disease might mimic other non-neoplastic oral lesions, which leads to a significant delay in diagnosing patients with OSCC. Thus, patients tend to present with late-stage OSCC, which is associated with metastatic phenotypes of oral cancer [2]. Moreover, cancers of the oral cavity and pharynx are continuously on the rise. Worldwide, oral cancers have an incidence of approximately 452,000 with an estimated mortality rate of 194,000 annually [3].

The current methods used to assess the prognosis of OSCC rely on the histopathological grading and TNM classification. These methods have been critically examined, and there are some limitations in their ability to assess prognosis accurately, especially for individual patients [4]. Therefore, novel approaches, such as the use of predictive protein biomarkers, are being explored to be used in conjunction with the current TNM classification and histopathological grading to help improve prognostic assessments.

Molecular biomarkers have a valuable role in cancer clinical management, including screening, risk stratification, and prognostic evaluation [5]. The increased prevalence of oral cancer highlights the need for molecular biomarkers to serve as diagnostic tools and prognosticators for disease progression assessment [6]. This study examines the differential expression of Serpin B5 as it correlates with diagnostic and prognostic parameters of oral cancer.

Serpin B5 is a member of the serine protease inhibitors superfamily and is encoded by a gene located on chromosome 18q21.3-q23 [7]. The 42-kDA protein is localized to epithelial cells in various tissues, including the squamous epithelium of the oral cavity [7]. It has been implicated in numerous cellular functions, including adhering to extracellular matrix components and contributing to cellular proliferation, migration, anti-angiogenesis, and invasion [7-14]. Although Serpin B5 expression has been associated with several other malignancies of squamous epithelial origin, studies have reported inconsistent expression patterns, particularly in OSCC [15-18]. Specifically, there have been several studies reporting an upregulation of Serpin B5 in OSCC that correlates with poorer prognosis [19,20], whereas other studies show that the downregulation of its expression indicates worse survival rates [21-23].

OSCC is a cancer that is continuously on the rise, with frequently missed early detection opportunities. Therefore, we aimed to characterize Serpin B5 as a molecular biomarker that can potentially aid in monitoring disease progression and predicting clinical outcomes by examining Serpin B5 expression levels as they correlate with histopathological grading and clinical staging, as indicated by the TNM classification based on tumor size, lymph node involvement, and metastasis, using immunohistochemical detection assay coupled with computer-assisted quantitative analysis. Our findings suggest that Serpin B5 is a promising molecular biomarker that can be useful in the detection and prognostic evaluation of patients with oral cancer.

## Materials and methods

Tissue samples

The tissue samples used for this study consisted of de-identified tissue microarray samples procured from TissueArray.com (Derwood, MD, USA) and Pantomics (Fairfield, CA, USA). A total of 183 samples were used in our analysis, including 12 samples representing normal oral mucosa and 171 samples representing OSCC with variable histopathological grading and TNM classification parameters. During analysis, the experimenters were blinded to all characteristics of the tissue samples.

Immunohistochemistry staining

The tissue samples were deparaffinized before they underwent immunohistochemistry (IHC) labeling with a 1:500 diluted Serpin B5 rabbit polyclonal primary antibody (Proteintech, Rosemont, IL, USA). The samples were incubated with the primary antibody at 23 degrees Celsius for 90 minutes, followed by incubation with 1:1000 diluted anti-rabbit IgG peroxidase-conjugated secondary antibody (Sigma-Aldrich, St. Louis, MO, USA) at 23 degrees Celsius for 60 minutes. Subsequently, immunoreactivity of the samples was visualized with 3,3’-diaminobenzidine (DAB) chromogenic substrate, followed by counterstaining with hematoxylin and sealing with coverslips for preservation.

Quantitative analysis of Serpin B5 expression

High-resolution images of the IHC-stained tissue samples were acquired with a high-resolution Nikon A1R inverted microscope (Nikon Corp., Tokyo, JPN) at 20x magnification. The microscope underwent optimization and standardization prior to formal imaging. Images were taken in one session to avoid alteration of microscope settings. Following image capture, computer-assisted image analysis was performed using Aperio ImageScope software (Leica Biosystems Inc., Buffalo Grove, IL, USA) to avoid the subjectivity and interobserver variability that are typically associated with IHC staining visual assessment.

Subsequently, a positive pixel counting algorithm was used to quantify the immunoreactivity of the tissues of interest. Samples underwent manual selection of regions of interest to ensure that only the tumor parenchyma and normal oral mucosa were included in the quantitative analysis and that the surrounding stroma and adjoining connective tissues were excluded. Immunoreactivity was determined based on the percentage of positively stained epithelial cells and staining intensity. The Histo-score (H-score) was generated by calculating the percentage of positively stained epithelial cells and the staining intensity. This included strong, medium, and weak positive pixels, which were assigned by red, orange, and yellow colors, respectively. For each set of clinicopathological parameters, the H-score was calculated using the formula: H-score = (1 x % weak positivity) + (2 x % medium positivity) + (3 x % strong positivity).

Statistical analysis

Tissue samples were categorized based on clinicopathological parameters before the H-scores were averaged for each respective group. The standard error of the mean (SEM) was calculated for each group of samples and then an unpaired t-test analysis was applied to each of the two compared groups using the GraphPad software (GraphPad Software Inc., San Diego, CA, USA) to determine statistical significance. The p-value was generated, and p < 0.05 was set as the threshold for statistically significant comparisons.

## Results

Serpin B5 as a diagnostic biomarker for OSCC

Using a combination of immunohistochemical detection and computer-assisted image analysis, a significant increase in Serpin B5 expression was documented in OSCC tissue samples compared to the normal oral mucosa (Figure 1).

**Figure 1:**
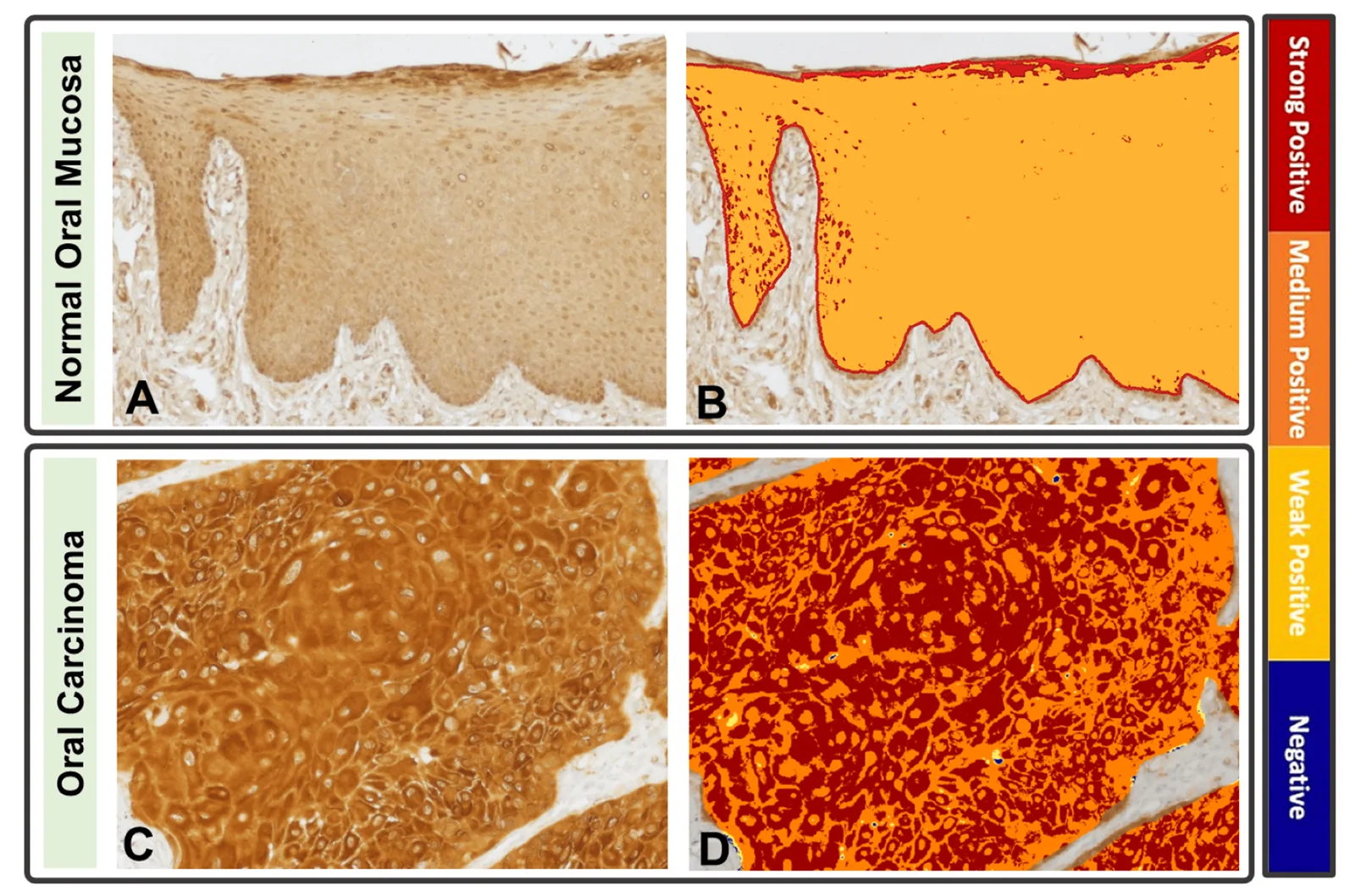
Serpin B5 expression in OSCC tissue samples in comparison to normal oral mucosa (IHC, 200x) The upper panel shows representative images of a normal oral mucosa tissue sample: (A) IHC with brown staining indicating immunoreactivity for Serpin B5 and (B) ImageScope computer-assisted analysis. The lower panel shows representative images of an OSCC sample: (C) IHC with brown staining indicating immunoreactivity for Serpin B5 and (D) ImageScope computer-assisted analysis. OSCC, oral squamous cell carcinoma; IHC, immunohistochemistry

Quantitative analysis of normal oral mucosa samples (n=12) yielded an average Histo-score of 1.2, while OSCC tissue samples (n=171) had an average Histo-score of 2. The upregulation of Serpin B5 expression in OSCC tissues proved to be statistically significant with a p-value of 0.0001 (Figure 2).

**Figure 2:**
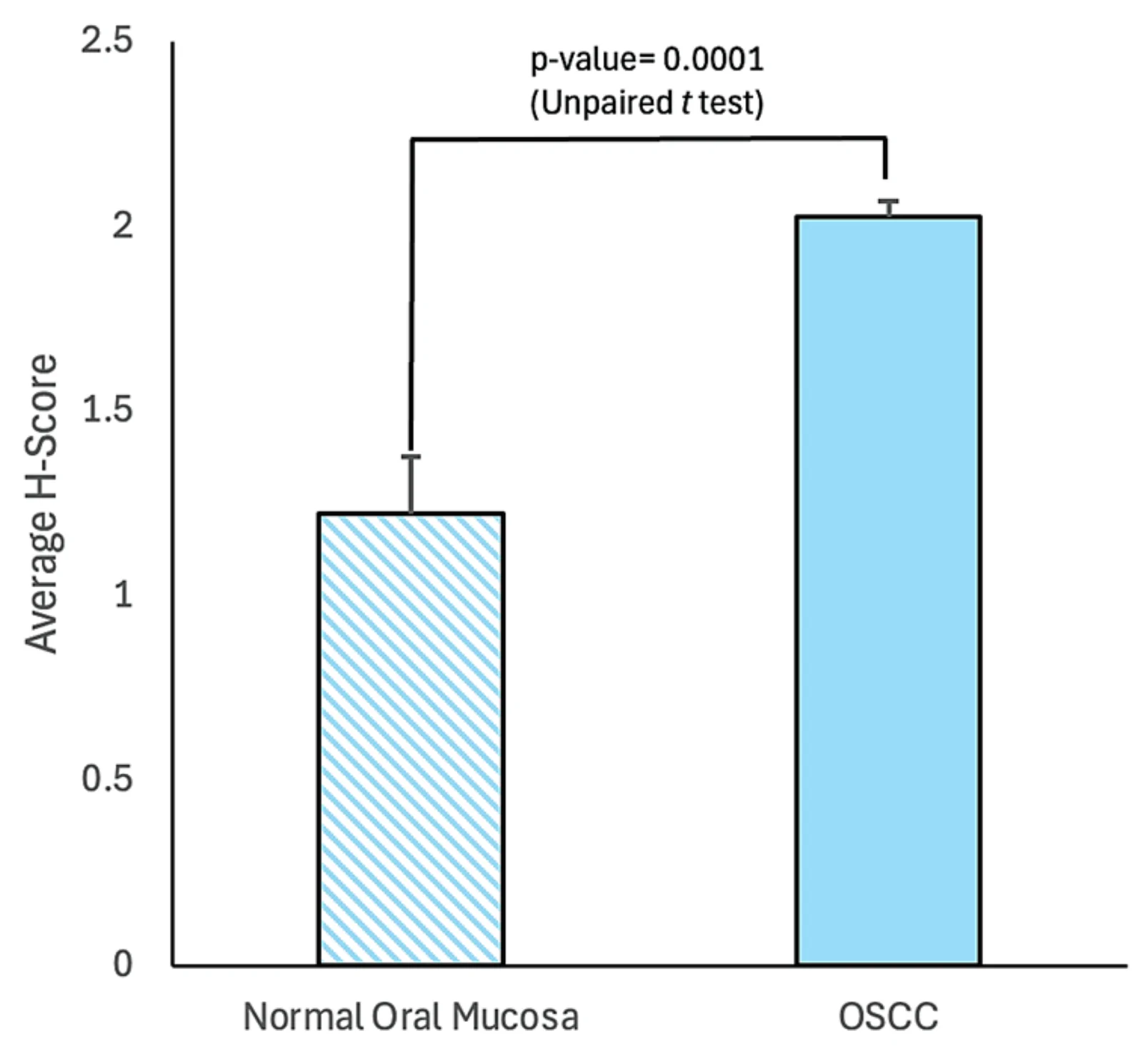
Average H-score as a measurement of Serpin B5 expression in OSCC in comparison to normal oral mucosa The bar graph depicts comparison of the average H-score for Serpin B5 immunoreactivity in normal oral mucosa samples (n=12) to OSCC samples (n= 171). An unpaired t-test was used for the comparison, the error bars represent the SEM, and a p-value of <0.05 is considered significant. H-score, Histo-score; OSCC, oral squamous cell carcinoma; SEM, standard error of the mean

Correlation between Serpin B5 expression and OSCC nodal status

Serpin B5 immunohistochemical detection coupled with computer-assisted image analysis of OSCC tissue samples with lymph node involvement (N1, N2, N3) demonstrated a decrease in Serpin B5 expression when compared to OSCC samples without any nodal metastases (N0) (Figure 3).

**Figure 3:**
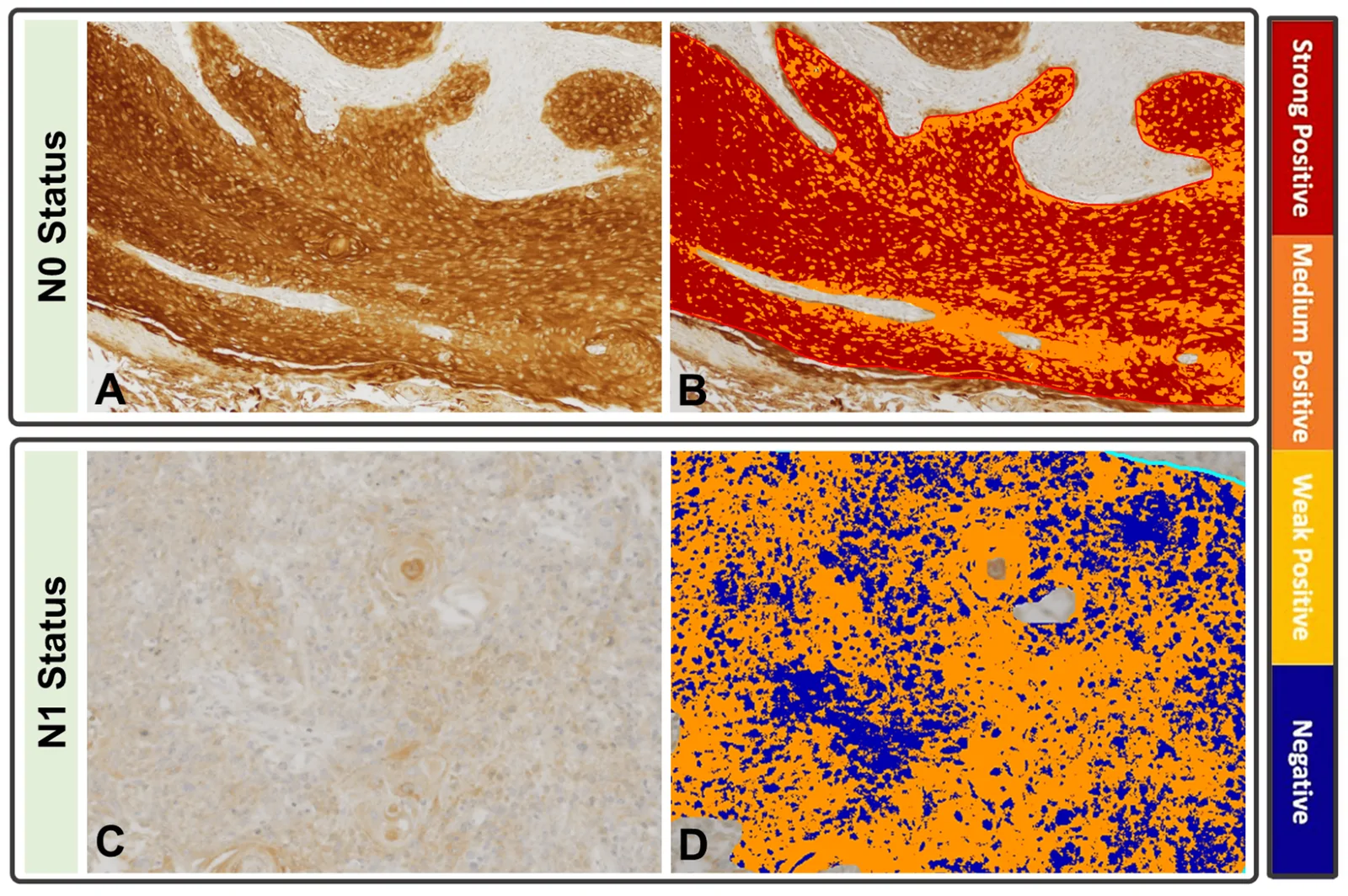
Serpin B5 expression in OSCC tissue samples with variable nodal status (IHC, 200x) The upper panel displays representative images of an OSCC tissue sample without nodal involvement (N0): (A) IHC with brown staining indicating immunoreactivity for Serpin B5 and (B) ImageScope computer–assisted analysis. the lower panel shows representative images of OSCC tissue samples with nodal status N1: (C) IHC with brown staining indicating immunoreactivity for Serpin B5 and (D) ImageScope computer-assisted analysis. OSCC, oral squamous cell carcinoma; IHC, immunohistochemistry

The average Histo-score for Serpin B5 immunoreactivity for a tissue sample with nodal involvement (N1, N2, N3) was 1.9 (n=56), while the average Histo-score of a sample without nodal involvement (N0) was 2.1 (n=113). The correlation of Serpin B5 expression with lymph node metastases was found to be statistically significant, with a p-value of 0.006 (Figure 4).

**Figure 4:**
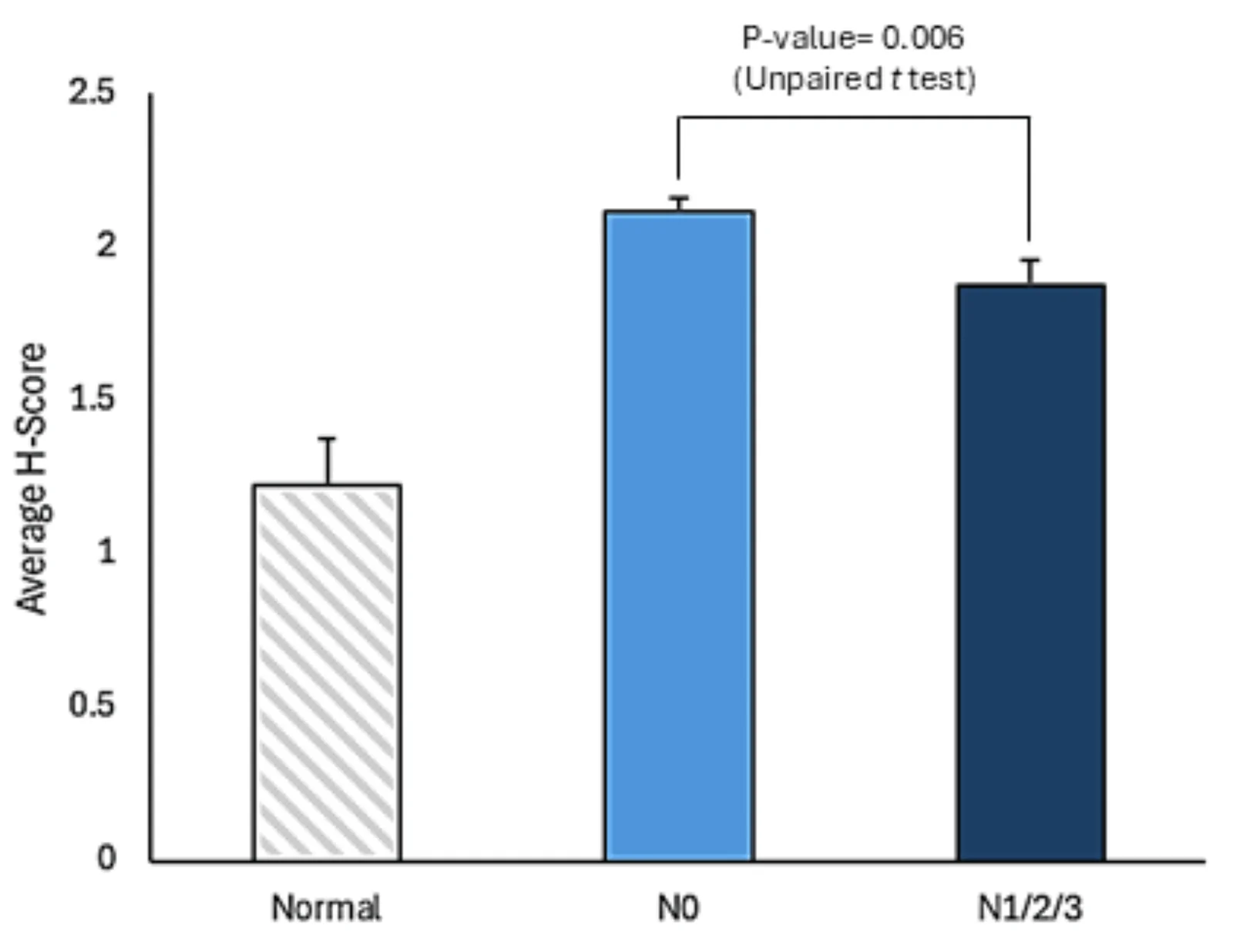
Average H-score as a measurement for Serpin B5 expression in OSCC with and without nodal metastases The bar graph depicts the comparison of the average H-score for Serpin B5 in samples of OSCC (n= 56) without nodal involvement (N0) to OSCC samples (n= 113) with nodal metastases (N1, N2, N3). The average H-score for the normal oral mucosa is shown as a reference only. An unpaired t-test was used for the comparison, the error bars represent the SEM, and a p-value of <0.05 is considered significant. H-score, Histo-score; OSCC, oral squamous cell carcinoma; SEM, standard error of the mean

Serpin B5 expression correlation with OSCC clinical staging

Serpin B5 immunoreactivity in combination with computer-assisted image analysis of OSCC tissue samples from patients with late clinical stages (stage III/IV) revealed a decrease in expression compared to OSCC tissue samples from patients with early clinical stages (stage I/II) (Figure 5).

**Figure 5:**
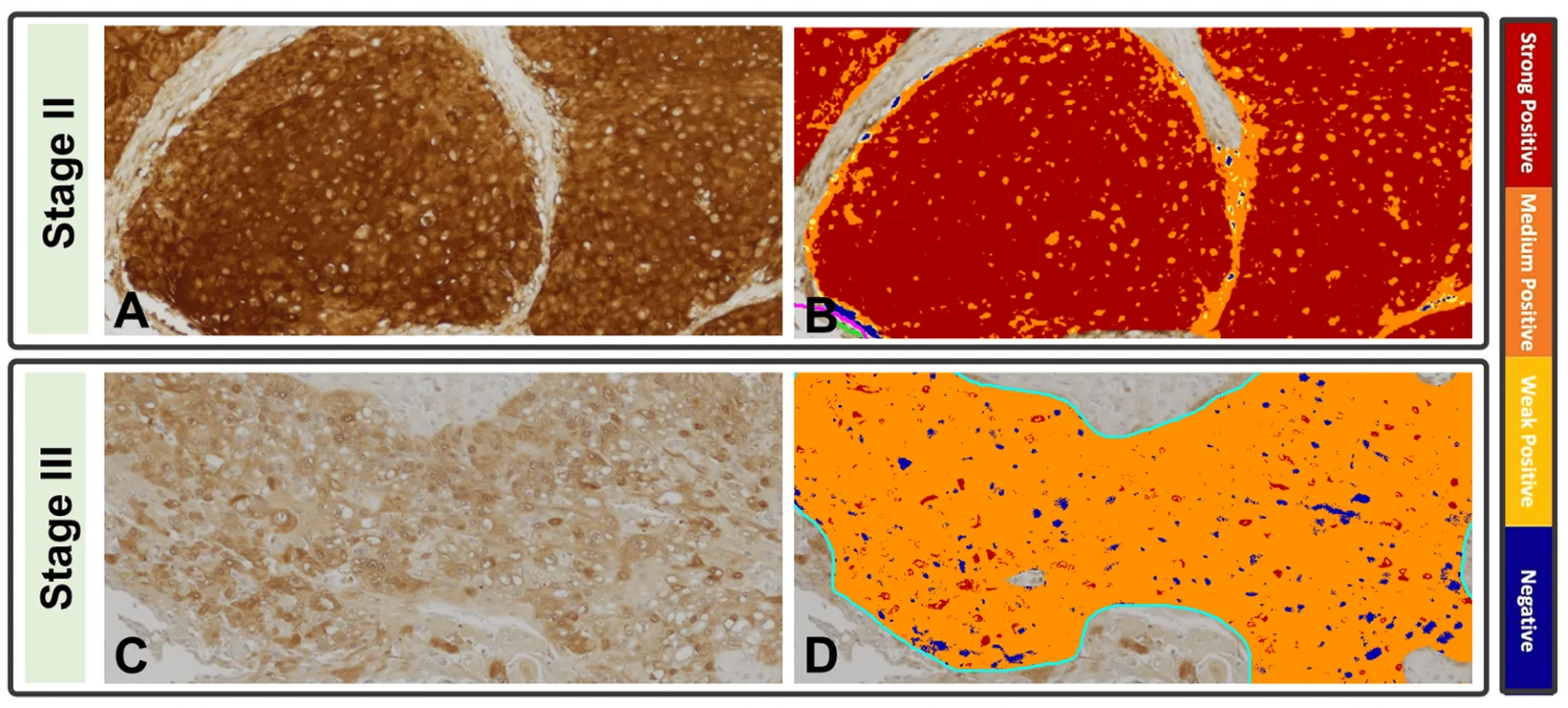
Serpin B5 expression in early clinical stages (I/II) in comparison to late clinical stages (III/IV) of OSCC (IHC, 200x) The upper panel shows representative images of an early-stage (stage II) OSCC tissue sample: (A) IHC with brown staining depicting immunoreactivity for Serpin B5 and (B) ImageScope computer-assisted analysis. The lower panel shows representative images of a late-stage (stage III) OSCC tissue sample: (C) IHC with brown staining depicting immunoreactivity for Serpin B5 and (D) ImageScope computer-assisted analysis. OSCC, oral squamous cell carcinoma; IHC, immunohistochemistry

The average Histo-score for Serpin B5 immunoreactivity in OSCC tissue samples from patients with early clinical stages (stages I/II) was 2.1 (n=85), while the average Histo-score for OSCC tissue samples from patients with late clinical stages (stages III/IV) was 1.9 (n=118). This correlation proved to be statistically significant with a p-value of 0.03 (Figure 6).

**Figure 6:**
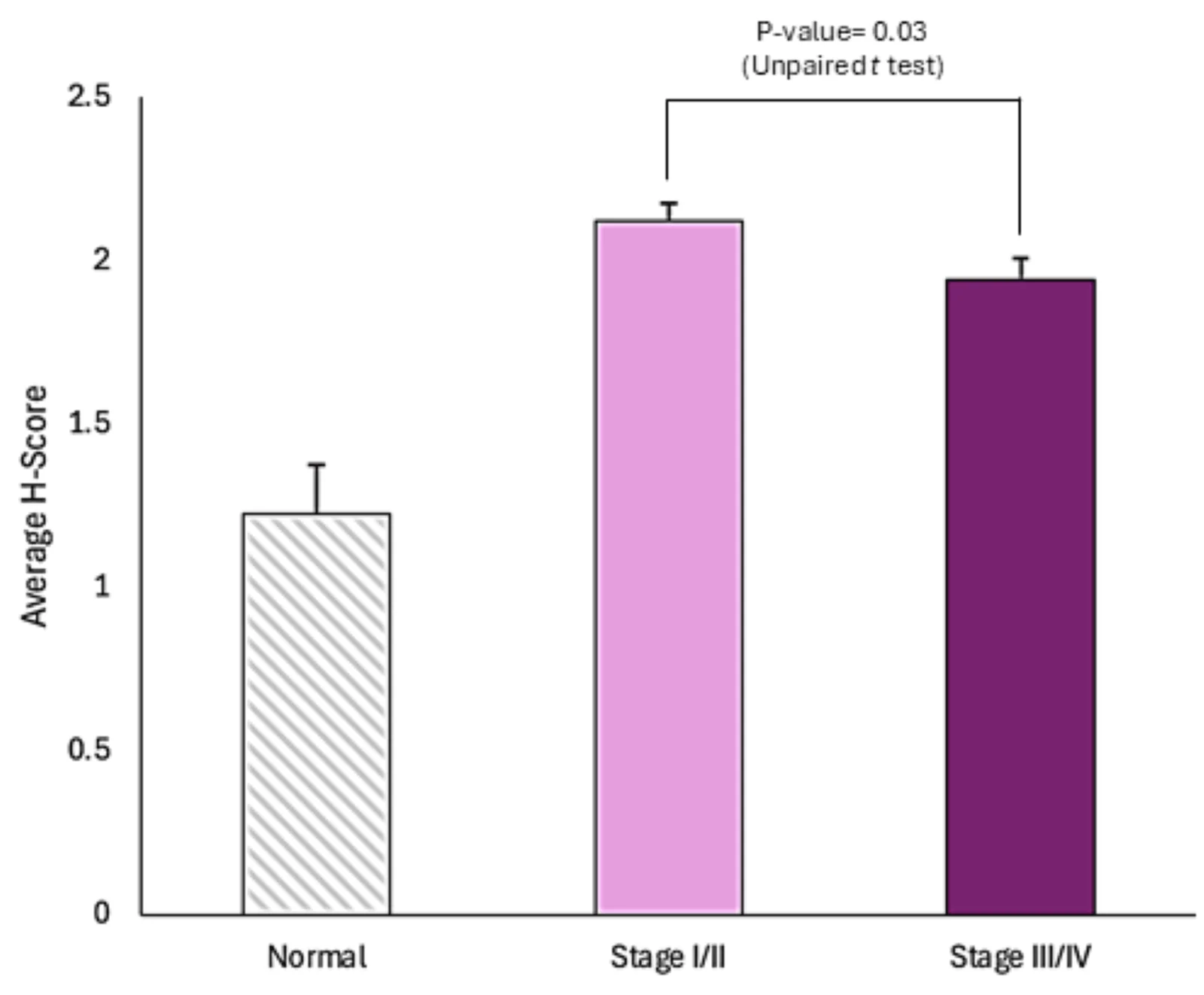
Average H-score as a measurement for Serpin B5 expression in OSCC samples in early clinical stages (stages I and II) in comparison to OSCC samples in later clinical stages (stages III and IV) The bar graph depicts the average H-score for Serpin B5 immunoreactivity in samples of OSCC of early clinical stages (stages I and II) compared to samples of OSCC with later clinical stages (stages III and IV). The average H-score for the normal mucosa is shown as a reference only. An unpaired t-test was used for the comparison, the error bars represent the SEM, and a p-value of <0.05 is considered significant. H-score, Histo-score; OSCC, oral squamous cell carcinoma; SEM, standard error of the mean

Serpin B5 expression correlation with OSCC histopathological grade/differentiation status

Quantitative analysis revealed a decreased Serpin B5 expression in high histopathological grade OSCC tissue samples (grade 2 and grade 3) in comparison to low histopathological grade OSCC tissue samples (grade 1) (Figure 7).

**Figure 7:**
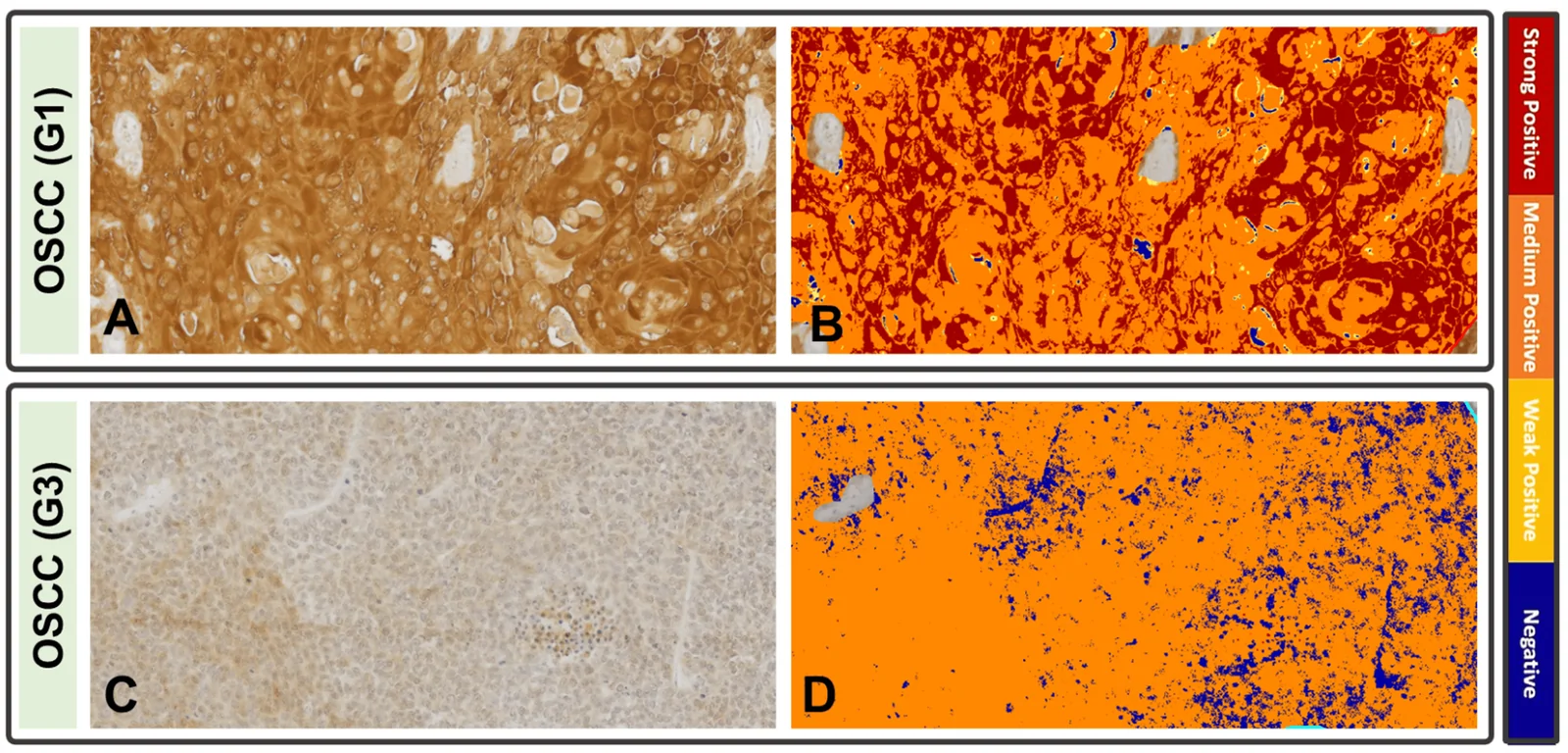
Serpin B5 expression in OSCC tissue samples with low histopathological grade in comparison to OSCC tissue samples with high histopathological grades (IHC, 200x) The upper panel shows representative images of an OSCC tissue sample with a low histopathological grade (G1): (A) IHC with brown staining depicting immunoreactivity for Serpin B5 and (B) ImageScope computer-assisted analysis. The lower panel shows representative images of an OSCC sample with a high histopathological grade (G3): (C) IHC with brown staining depicting immunoreactivity for Serpin B5 and (D) ImageScope computer-assisted analysis. OSCC, oral squamous cell carcinoma; IHC, immunohistochemistry

OSCC tissue samples with low histopathological grade (G1) scored an average Histo-score of 2.1 (n=88), while the average Histo-score from OSCC tissue samples with high histopathological grade (G2, G3) was 1.8 (n=71). The correlation between Serpin B5 expression and histopathological grading, which indicates the differentiation status of a malignant neoplasm, demonstrated statistical significance with a p-value of 0.0008 (Figure 8).

**Figure 8:**
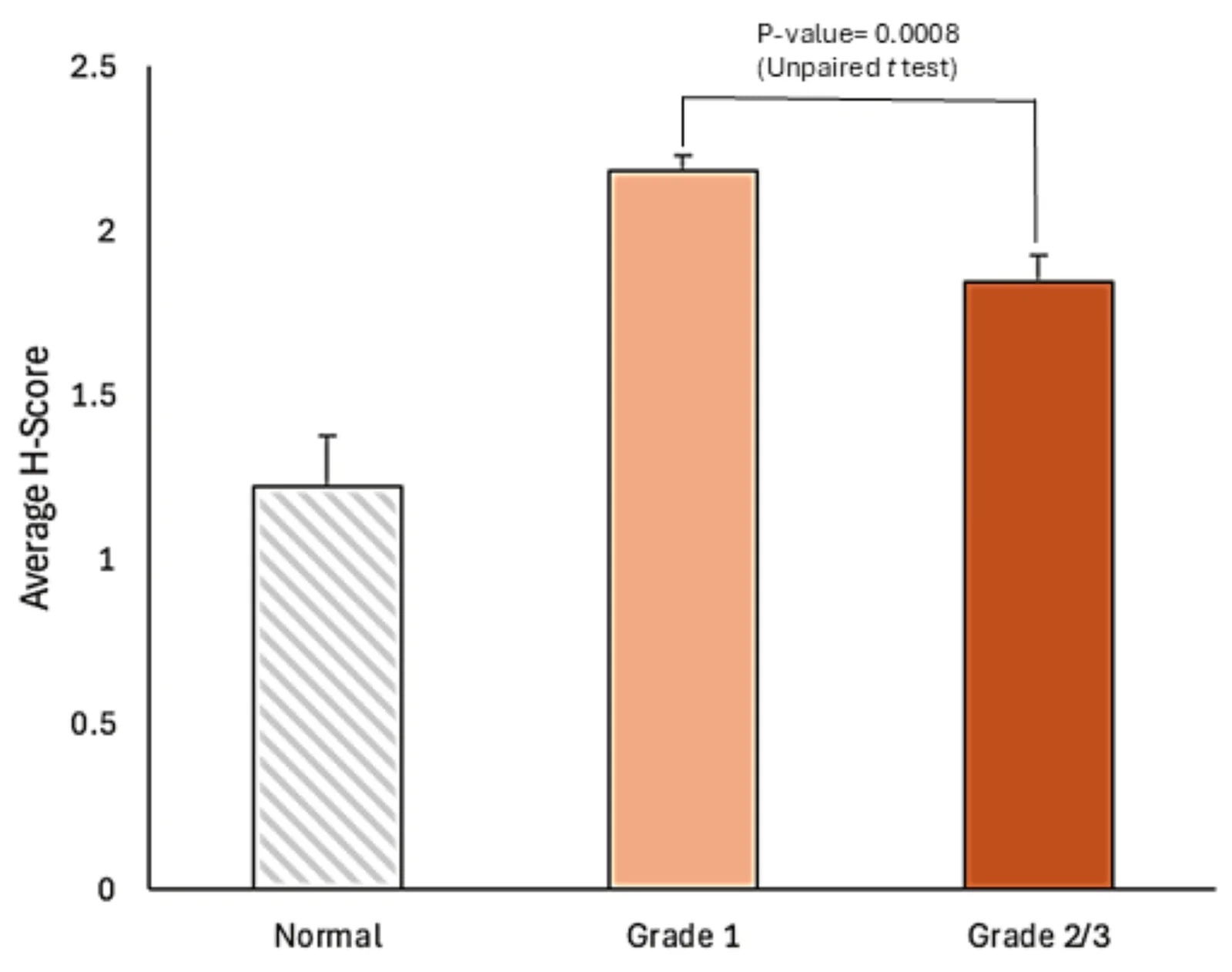
Average H-score as a measurement for Serpin B5 expression in OSCC samples with low histopathological grade in comparison to OSCC samples with high histopathological grades The bar graph depicts the average H-score for Serpin B5 immunoreactivity in samples of OSCC with low histopathological grade (G1) compared to OSCC samples with high histopathological grades (G2 and G3). The average H-score for the normal mucosa is shown as a reference only. An unpaired t-test was used for the comparison, the error bars represent the standard error of the mean (SEM), and a p-value of <0.05 is considered significant. H-score, Histo-score; OSCC, oral squamous cell carcinoma; SEM, standard error of the mean

## Discussion

The protein Serpin B5 is involved in several cellular and neoplastic processes, including cell adhesion, proliferation, migration, and invasion [7-14,24]. While prior studies sought to document the expression of Serpin B5 in cancer tissues, its overall expression trends have remained largely elusive, especially when it comes to its association with clinical parameters and outcomes [19-23]. This study explored the potential role of Serpin B5 as a diagnostic and prognostic biomarker for OSCC and documented a significant upregulation of Serpin B5 expression in OSCC tissue samples compared to normal counterpart tissues.

The study also found that Serpin B5 was significantly decreased in OSCC samples with involvement of the lymph nodes compared to OSCC samples without nodal involvement. Additionally, OSCC samples from patients with advanced clinical stages (stages III and IV) had decreased expression of Serpin B5 in comparison to those from patients with early clinical stages (stages I and II). Moreover, the less differentiated OSCC tissue samples with higher histopathological grades (G2, G3) had decreased Serpin B5 expression compared to the more differentiated OSCC tissue samples with low histopathological grade (G1).

These findings are clinically relevant as early detection is crucial for improved clinical outcomes. The localized disease has a five-year relative survival rate of 88.7%; however, only approximately 25% of cases are diagnosed at this stage [1]. In metastatic OSCC, the relative survival rate drops to approximately 36% [1]. Therefore, early detection is critical as it allows for improved survival and quality of life [4]. Our results demonstrate Serpin B5 expression correlation with the critical prognostic indicators of TNM clinical staging and histopathological grading, which suggests that Serpin B5 expression analysis could be used as a supplementary tool to aid in the detection of oral cancers at earlier stages and monitor disease progression and therapeutic responses throughout the course of the disease. We envision that Serpin B5 can be used to supplement existing biomarkers, such as cytokeratins, to enhance their predictive power.

The limitations of this study are primarily related to the use of tissue microarrays, where the small cores of tissue may not be truly representative of whole tumors, despite the advantage of processing multiple samples for biomarker discovery in a rapid, uniform manner. Additionally, tissue microarrays tend to be archival, resulting in a lack of real-time clinical data such as survival and disease recurrence rates. Overall, the retrospective study design has limited the scope of the study to a certain extent. Therefore, follow-up studies will be needed to document Serpin B5 expression patterns in a large cohort of oral cancer patients and to correlate expression levels with relative survival and recurrence rates to further substantiate Serpin B5 as a biomarker for oral cancer in the clinical setting.

## Conclusions

Serpin B5 demonstrated a significant upregulation in expression in oral cancer compared to its tissue of origin in the normal oral mucosa. This is clinically relevant as it highlights its applicability as a clinical diagnostic tool. The expression of Serpin B5 was significantly downregulated with nodal metastases as well as with advanced clinical stages, which are directly correlated with survival rates. Furthermore, Serpin B5 expression was significantly downregulated in tissue samples with high histopathological grades, which are typically associated with a more aggressive behavior in tumors. This study is retrospective by design; however, it underscores the potential role of Serpin B5 as a supplementary tool to aid in evaluating and monitoring disease progression in patients with oral cancer. Subsequent prospective longitudinal studies examining larger cohorts of patients with survival and therapeutic outcome follow-up clinical data are needed.
